# Identification of blood exosomal metabolomic profiling for high-altitude cerebral edema

**DOI:** 10.1038/s41598-024-62360-0

**Published:** 2024-05-21

**Authors:** Quan Tang, Fangcheng Fan, Lei Chen, Yuewen Chen, Lin Yuan, Lili Wang, Huan Xu, Yan Zhang, Yong Cheng

**Affiliations:** 1 Key Laboratory of Mass Spectrometry Imaging and Metabolomics, Center on Translational Neuroscience, College of Life and Environmental Sciences, Minzu University of China, Beijing, China; 2 Key Laboratory of Ethnomedicine of Ministry of Education, School of Pharmacy, Minzu University of China, Beijing, China; 3 Chinese Academy of Sciences Key Laboratory of Brain Connectome and Manipulation, Shenzhen Key Laboratory of Translational Research for Brain Diseases, The Brain Cognition and Brain Disease Institute, Shenzhen Institute of Advanced Technology, Chinese Academy of Sciences; Shenzhen–Hong Kong Institute of BrainScience—Shenzhen Fundamental Research Institutions, Shenzhen, China; 4 Guangdong Provincial Key Laboratory of Brain Science, Disease and Drug Development, HKUST Shenzhen Research Institute, Shenzhen, China; 5 Department of Clinical Laboratory, The General Hospital of Tibet Military Command, Lhasa, China; 6 Institute of National Security, Minzu University of China, Beijing, China

**Keywords:** High-altitude cerebral edema (HACE), Exosome, Metabolomics, Blood, Biomarkers, Hypoxic-ischaemic encephalopathy

## Abstract

High-altitude cerebral edema (HACE) is a severe neurological condition that can occur at high altitudes. It is characterized by the accumulation of fluid in the brain, leading to a range of symptoms, including severe headache, confusion, loss of coordination, and even coma and death. Exosomes play a crucial role in intercellular communication, and their contents have been found to change in various diseases. This study analyzed the metabolomic characteristics of blood exosomes from HACE patients compared to those from healthy controls (HCs) with the aim of identifying specific metabolites or metabolic pathways associated with the development of HACE conditions. A total of 21 HACE patients and 21 healthy controls were recruited for this study. Comprehensive metabolomic profiling of the serum exosome samples was conducted using ultraperformance liquid chromatography–tandem mass spectrometry (UPLC‒MS/MS). Additionally, Kyoto Encyclopedia of Genes and Genomes (KEGG) pathway enrichment analysis was performed to identify the metabolic pathways affected in HACE patients. Twenty-six metabolites, including ( +)-camphoric acid, choline, adenosine, adenosine 5′-monophosphate, deoxyguanosine 5′-monophosphate, guanosine, and hypoxanthine-9-β-D-arabinofuranoside, among others, exhibited significant changes in expression in HACE patients compared to HCs. Additionally, these differentially abundant metabolites were confirmed to be potential biomarkers for HACE. KEGG pathway enrichment analysis revealed several pathways that significantly affect energy metabolism regulation (such as purine metabolism, thermogenesis, and nucleotide metabolism), estrogen-related pathways (the estrogen signaling pathway, GnRH signaling pathway, and GnRH pathway), cyclic nucleotide signaling pathways (the cGMP-PKG signaling pathway and cAMP signaling pathway), and hormone synthesis and secretion pathways (renin secretion, parathyroid hormone synthesis, secretion and action, and aldosterone synthesis and secretion). In patients with HACE, adenosine, guanosine, and hypoxanthine-9-β-D-arabinofuranoside were negatively correlated with height. Deoxyguanosine 5′-monophosphate is negatively correlated with weight and BMI. Additionally, LPE (18:2/0:0) and pregnanetriol were positively correlated with age. This study identified potential biomarkers for HACE and provided valuable insights into the underlying metabolic mechanisms of this disease. These findings may lead to potential targets for early diagnosis and therapeutic intervention in HACE patients.

## Introduction

High-altitude cerebral edema (HACE) is a rare and potentially fatal manifestation of acute mountain sickness (AMS)^1^. HACE syndrome is considered to indicate malignant progression of AMS^2^ and is influenced by factors such as altitude, the rate of ascent, the duration of exposure to high altitude, infection, mood, and genetic predisposition^3,4^. The rapid onset and progression of HACE can lead to severe sequelae; therefore, it is imperative to understand the mechanisms of HACE for early treatment and improved prognosis.

HACE syndrome is characterized by symptoms of AMS accompanied by ataxia or changes in mental status^5^. The potential mechanisms of HACE include vascular leakage resulting in extracellular edema due to increased blood‒brain barrier permeability, as well as cytotoxic edema within the cells^6^. Hypoxia plays a crucial role in the development of HACE, with hypoxemia leading to cerebral vasodilation, increased cerebral blood flow, and dysfunction of the blood–brain barrier, resulting in leakage and vasogenic cerebral edema. These mechanisms contribute to the neurological manifestations of HACE^7,8^. Additionally, the severity and occurrence of HACE are influenced by functional polymorphisms of key enzymes involved in physiological pathways^9^.

Exosomes are small extracellular vesicles with diameters between 30 and 150 nm, which play crucial roles in cell-to-cell communication, are enriched in proteins and lipids^10^. These small vesicles have gained attention in recent years for their involvement in disease-specific metabolic profiles and their potential applications in early disease diagnosis, monitoring disease status, and drug delivery^11,12^. Moreover, exosomes have been found to cross the BBB^13^, suggesting that they are intriguing targets for investigating physiological changes and identifying metabolic alterations in HACE. In this study, we utilized ultraperformance liquid chromatography–tandem mass spectrometry (UPLC‒MS/MS) to analyze the metabolomic characteristics of blood exosomes from HACE patients, with the aim of identifying metabolic changes associated with HACE and providing new insights into the physiological mechanisms, early diagnosis, and potential drug targets of HACE.

## Materials and methods

### Participants

A total of 21 HACE patients diagnosed by professional doctors were recruited from The General Hospital of Tibet Military Command. Twenty-one healthy individuals were recruited as healthy control (HC) participants, and the HACE patients were matched in terms of age and sex. The main characteristics of all participants are shown in Table 1. Written informed consent was obtained from all participants. The research protocol was approved by the Ethics Committee of Minzu University of China, Beijing, China, and this study was conducted in accordance with the Declaration of Helsinki.

**Table 1:** Demographic characteristics of the subjects.

HACE (High altitude cerebral edema)							HC (Healthy Controls)		
Name	Age	Gender	Height	Weight	BMI	Outcome	Name	Age	Gender
CE1	31	Male	180	76	23.4568	Survival	HC1	49	Male
CE2	43	Male	175	80	26.1224	Survival	HC2	40	Male
CE3	45	Male	165	NA	NA	Death	HC3	39	Male
CE4	33	Male	170	90	31.1419	Survival	HC4	37	Male
CE5	36	Male	170	60	20.7612	Survival	HC5	36	Male
CE6	26	Male	172	65	21.9713	Survival	HC6	35	Male
CE7	24	Male	175	NA	NA	Survival	HC7	35	Male
CE8	36	Male	170	60	20.7612	Survival	HC8	34	Male
CE9	29	Male	NA	NA	NA	Survival	HC9	34	Male
CE10	31	Male	173	79	26.3958	Survival	HC10	31	Male
CE11	26	Female	160	60	23.4375	Survival	HC11	25	Male
CE12	54	Male	NA	NA	NA	Survival	HC12	25	Male
CE13	45	Male	178	80	25.2493	Survival	HC13	25	Male
CE14	22	Female	168	70	24.8016	Survival	HC14	25	Male
CE15	45	Male	171	60	20.5191	Survival	HC15	24	Male
CE16	37	Male	NA	NA	NA	Survival	HC16	24	Male
CE17	47	Male	170	75	25.9516	Survival	HC17	49	Male
CE18	50	Male	NA	NA	NA	Survival	HC18	30	Male
CE19	50	Male	165	70	25.7117	Survival	HC19	23	Female
CE20	67	Male	NA	NA	NA	Survival	HC20	27	Female
CE21	35	Male	167	100	35.8564	Survival	HC21	42	Male
	38.67 ± 11.38							32.81 ± 8.00	

BMI, Body mass index; NA, Not available.

### Exosome isolation

Blood samples were collected from both the control and HACE participants following an overnight fast. After allowing the blood to clot at room temperature for 2 h, the serum was obtained by centrifuging at 3000×*g* for 15 min. The serum was then stored at − 80 °C until further analysis. Serum exosomes were isolated using 70 nm qEVoriginal Columns method provided by Izon (Ser. #: 1,004,555, Oxford, UK). Subsequently, the collected exosomes were concentrated by centrifugation at 5000×*g* for 10 min using the 30 K MWCO PES protein concentrator Vivaspin (Sarorius, Goettingen, Germany). The concentrated exosomes were resuspended in 100 μl of PBS for subsequent analysis.

### Western blot analysis

Protein quantification was performed using the BCA Protein Assay Kit (P0011, Beyotime, Shanghai, China). Sample proteins were loaded onto a 10% SDS-PAGE gel, electrophoresed, and transferred to a PVDF membrane (0.45 µm; Millipore, Billerica, MA, USA). After blocking with 5% non-fat milk, the membrane was probed with specific antibodies overnight at 4 ℃, including anti-CD63 (A5271, ABclonal, Wuhan, China) and anti-TSG101 (A1692, ABclonal, Wuhan, China). On the following day, the membranes were incubated with the appropriate secondary antibody (ZB-5301, ZSGB-BIO, Beijing, China) at room temperature for 1 h. Immunoblots were then visualized using the BeyoECL Plus Kit (P0018, Beyotime, Shanghai, China).

### Metabolite measurements

Comprehensive metabolomic profiling of serum exosome samples was conducted using UPLC‒MS/MS, as previously described^14^. Both first- and second-order mass spectrometry were employed for qualitative analysis utilizing a metabolomic data management system and public metabolite databases. The metabolites were measured using triple quadrupole mass spectrometry and a multiple-reaction monitoring method.

### Differential expression analysis

Orthogonal projections to latent structures discriminant analysis (OPLS-DA) was applied to assess the differential expression of metabolites. Variable importance in projection (VIP) values were extracted from the model^15^. Metabolites with VIP ≥ 1^16^ and an absolute Log_2_FC (fold change) ≥ 2, as determined by the Mann‒Whitney U test, were considered to be differentially expressed.

### Bioinformatics analysis

To gain insight into the biological functions of the identified differentially expressed metabolites, Kyoto Encyclopedia of Genes and Genomes (KEGG) pathway annotation was performed using MetaboAnalyst software^17^. The metabolic pathways with a significance level of *P* < 0.05 were considered significantly enriched.

### Statistical analysis

Unsupervised principal component analysis (PCA) was conducted using the prcomp statistical function in R software^18^. Receiver operating characteristic (ROC) curve analysis^17^ was employed to assess the ability of exosomal metabolites to distinguish between the HCs and HACE patients.

### Ethics approval and consent to participate

Written informed consent was obtained from all participants. The research protocol was approved by the Ethics Committee of Minzu University of China, Beijing, China, and this study was conducted in accordance with the Declaration of Helsinki.

## Results

The results of exosome identification are presented in Supplementary Fig. 1. Nanoparticle tracking analysis (NTA) revealed that the peak particle size of the extracted exosomes was 86.7 nm, with over 98% falling within the range of 30–150 nm (Supplementary Fig. 1A). Western blot analysis confirmed the presence of specific exosome markers—CD63 and TSG101 (Supplementary Fig. 1B). Additionally, the concentration of exosomes in serum samples was quantified using the BCA protein assay, as shown in Supplementary Fig. 2. There was no significant difference in serum exosome concentration between individuals with HC and HACE (2.472 ± 0.913 mg/mL, 2.284 ± 1.376 mg/mL; *P* = 0.6968). Furthermore, an analysis of blood parameters in individuals with HACE and HC revealed a significant increase in WBC, NEUT, MONO, and CRP levels in HACE compared to HC. Conversely, there was a notable decrease in RBC, LYM, and EO levels in individuals with HACE. Detailed data are provided in Supplementary Table 1.

**Figure 1:**
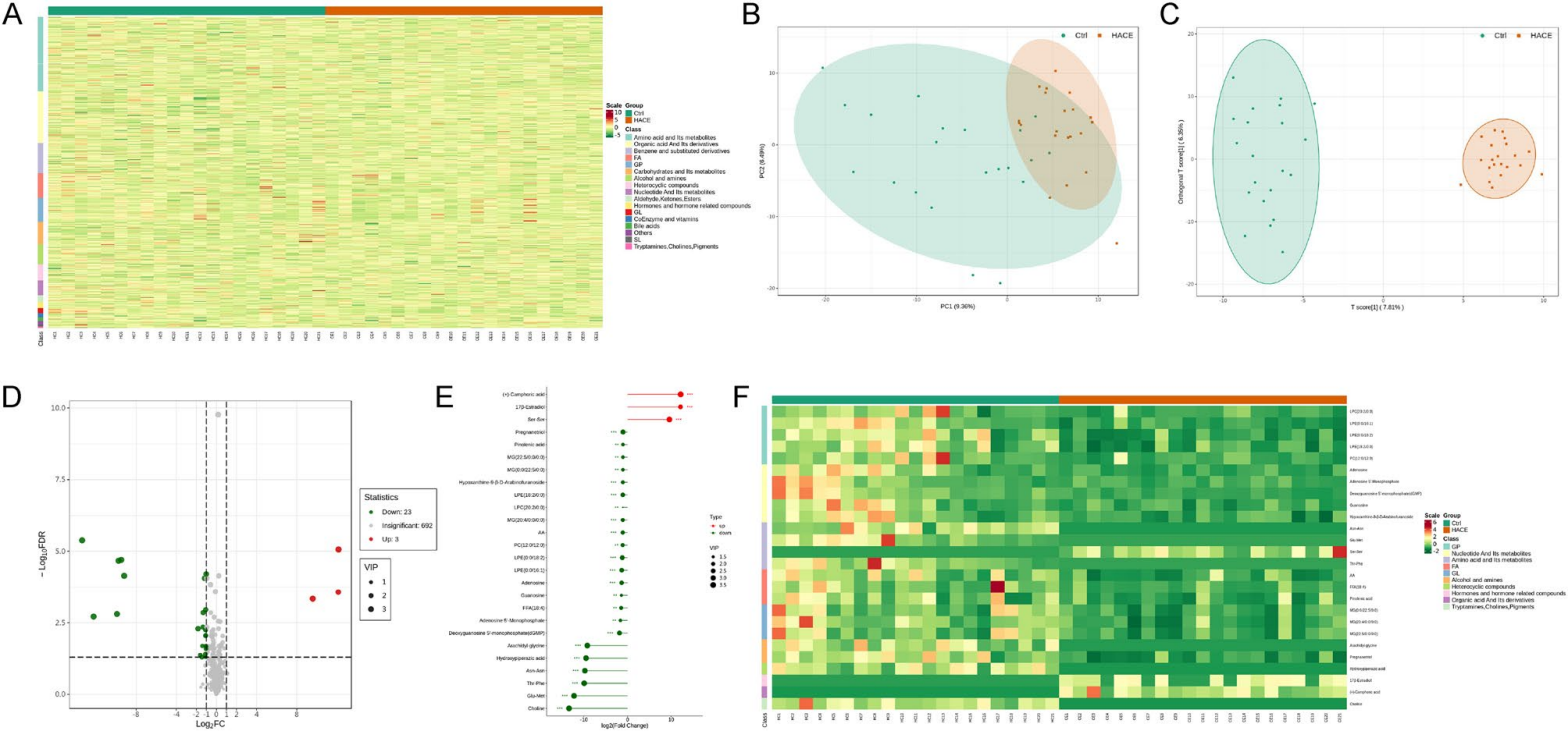
Bioinformatic screening for the differential expression of metabolites in blood exosomes of individuals with high-altitude cerebral edema (HACE). (**A**) Cluster heatmap illustrating all substances; (**B**) Principal component analysis (PCA) and (**C**) orthogonal partial least squares-discriminant analysis (OPLS-DA) model plot in the training participant set; (**D**) Volcano plot depicting differences in metabolites between HACE participants and healthy controls; (**E**) Cluster heatmap of differential substances.

The results of our study revealed significant differential expression of blood exosomal metabolites in HACE patients. We conducted a data normalization process, followed by cluster heatmap analysis of all the samples (Fig. 1A). Subsequently, PCA was performed on the samples grouped for differential comparison, revealing distinct metabolic profiles between the HACE and HC groups (Fig. 1B). An OPLS-DA model was applied to identify the differentially expressed metabolites between these two groups (Fig. 1C), revealing three upregulated and twenty-three downregulated metabolites in patients with HACE compared to HCs (Fig. 1D). Additionally, the fold changes in the quantitative information of the metabolites between the HC and HACE groups are depicted in Fig. 1E,F.

**Figure 2:**
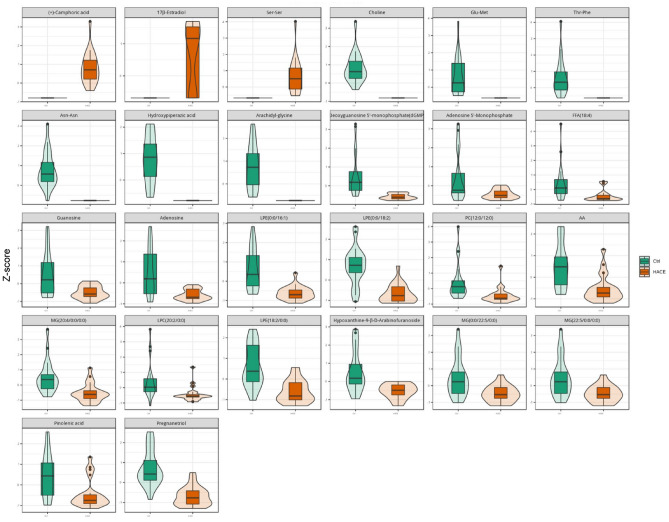
Violin plots showing the differential expression of blood exosomal metabolites in HACE patients.

**Figure 3:**
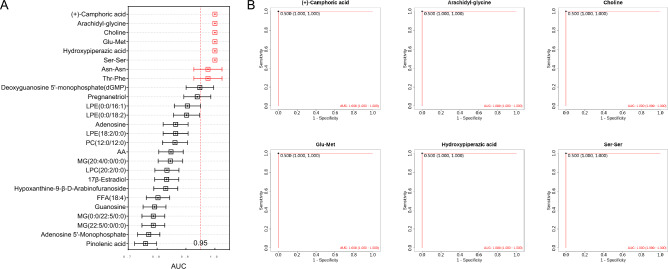
Blood exosomal metabolites as biomarkers for differentiating between HACE patients and controls (**A**) ROC curves used to evaluate the accuracy of a cluster of 26 metabolites for the diagnosis of HACE in a training set of participants; (**B**) ROC curve of 6 metabolites with full scores.

The differentially abundant metabolites, including ( +)-camphoric acid, choline, adenosine, adenosine 5′-monophosphate, deoxyguanosine 5′-monophosphate, guanosine, and hypoxanthine-9-β-D-arabinofuranoside, are visually presented (Fig. 2). Furthermore, this study explored the potential of exosomal metabolites as biomarkers for HACE. The analysis of the ROC curve using 26 exosomal metabolites revealed promising results in differentiating between HACE patients and HCs (Fig. 3).

**Figure 4:**
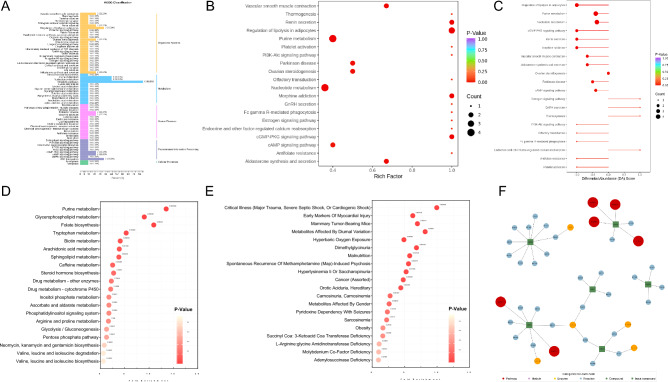
KEGG pathway enrichment analysis of differentially abundant metabolites. (**A**) Illustrated differentially abundant metabolite KEGG classification diagram; (**B**) enrichment map of differentially abundant metabolitesmetabolites according to KEGG enrichment; (**C**) difference abundance score chart; (**D**) MSEA enrichment analysis chart based on 84 KEGG metabolic sets (blood) (selected from the top 20 metabolic sets with *P* values are plotted); (**E**) MSEA enrichment analysis of differentially abundant metabolite pathway metabolic sets (selected from the top 20 metabolic sets with *P* values are plotted); (**F**) network diagram based on 339 blood regulatory pathways.

Based on the KEGG information of the differentially abundant metabolites identified using the aforementioned filtering criteria, KEGG metabolic pathways that contained at least one differentially abundant metabolite were selected. We subsequently classified metabolites that exhibited significant differences according to the pathway types in the KEGG database. Among them, there were three metabolic categories with three or more differentially abundant metabolites enriched in the KEGG pathway, concentrated in the energy metabolism pathway (Fig. 4A). The top 20 metabolic pathways related to purine metabolism, regulation of lipolysis in adipocytes, vascular smooth muscle contraction, thermogenesis, renin secretion, platelet activation, parathyroid hormone synthesis, secretion, and action were found to be significantly affected in the HACE group compared to the HCs. The difference abundance score plot revealed the downregulation of pathways related to the regulation of lipolysis in adipocytes, purine metabolism, nucleotide metabolism, the cGMP-PKG signaling pathway, and morphine addiction, while pathways related to GnRH secretion, endocrine and other factor-regulated calcium reabsorption, and the estrogen signaling pathway were upregulated in HACE patients compared to HCs (Fig. 4B,C).

MSEA enrichment analysis of 84 KEGG pathway metabolic sets and 339 exosomal metabolic sets enabled us to visualize the differentially abundant metabolites and conduct pathway searches and regulatory interaction network analysis (Fig. 4D,E). The network plot showed that 5′-nucleotidase (EC3.1.3.5) had the most common connections with the differentially abundant metabolites and played a crucial role in energy metabolism (Fig. 4F).

**Figure 5:**
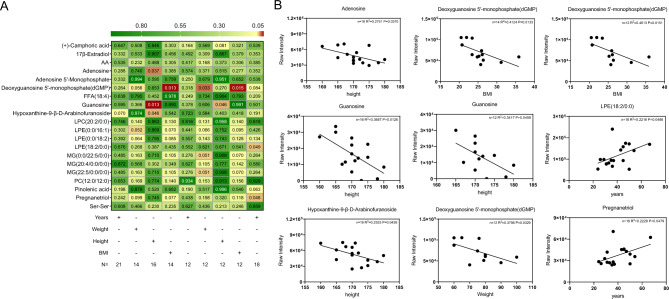
Pearson correlation analysis of various differentially abundant metabolite raw intensities with the height, BMI, weight, and age of HACE participants. (**A**) A heatmap displaying correlation *P* values; (**B**) 9 Correlation analysis diagrams of *P* values < 0.05.

The Pearson correlation analysis revealed several significant associations (Fig. 5A). Due to the inability to measure height and weight in some patients in a coma and bedridden state, there were fewer than 21 individuals in the correlation analysis of weight (n = 14), height (n = 16), and body mass index (BMI) (n = 14). Additionally, N = 12 and N = 18 indicate that the sample in this group was entirely male. Specifically, as shown in Fig. 5B, height exhibited an inverse relationship with adenosine (N = 16, R^2^ = 0.2751, *P* = 0.037), guanosine (N = 16, R^2^ = 0.3687, *P* = 0.0126; N = 12, R^2^ = 0.3417, *P* = 0.0459), and hypoxanthine-9-β-D-arabinofuranoside (N = 16, R^2^ = 0.2553, *P* = 0.0459). BMI was negatively correlated with deoxyguanosine 5′-monophosphate (n = 16, R^2^ = 0.4124, *P* = 0.0133; n = 12, R^2^ = 0.4613, *P* = 0.0151). Similarly, weight was negatively associated with deoxyguanosine 5’-monophosphate (n = 12, R^2^ = 0.3796, *P* = 0.0329). In contrast, the age of the HACE-related subjects was positively correlated with LPE (18:2/0:0) (n = 18, R^2^ = 0.2216, *P* = 0.0486) and Pregnanetriol (n = 18, R^2^ = 0.2228, *P* = 0.0479).

## Discussion

HACE is a condition characterized by cerebral edema and neurological impairment that occurs in low-pressure, hypoxic environments. It leads to damage to the endothelial cells of the blood‒brain barrier and results in abnormal metabolic phenomena^19^. Exosomes contain proteins, lipids, amino acids, and metabolites and play crucial roles in cell communication^20^. We hypothesized that HACE may cause functional changes in the brain, leading to alterations in blood exosome metabolism. To explore this phenomenon, we utilized UPLC‒MS/MS to analyze the metabolomic profile of blood exosomes in HACE. Our findings revealed differential expression of metabolites in the blood exosomes of individuals with HACE. Specifically, 29 metabolites, including ( +)-camphoric acid, choline, adenosine, adenosine 5′-monophosphate, deoxyguanosine 5′-monophosphate, guanosine, hypoxanthine-9-β-D-arabinofuranoside, and others, exhibited significant changes in expression in HACE patients compared to HCs. Furthermore, enrichment pathway analysis revealed several pathways, such as purine metabolism, regulation of lipolysis in adipocytes, vascular smooth muscle contraction, thermogenesis, renin secretion, platelet activation, parathyroid hormone synthesis, secretion, and action, that were significantly affected in HACE. Collectively, the data from this study indicate an imbalance in metabolite levels in the serum exosomes of individuals with HACE.

HACE is associated with prolonged exposure to hypobaric hypoxia in high-altitude environments^21^. Hypobaric hypoxia can cause severe brain damage and impair mitochondrial function, contributing to hypoxic brain injury^22^. Mitochondria primarily utilize inhaled oxygen for oxidative phosphorylation, which generates adenosine triphosphate (ATP). Hypoxia significantly affects mitochondria, jeopardizes cellular energy availability, damages mitochondrial components, alters mitochondrial quality and dynamics, and influences mitochondrial cell death pathways^23^. Our study identified differential expression profiles related to energy metabolism in the blood exosome-derived metabolites of individuals with HACE. Metabolites such as adenosine, adenosine 5′-monophosphate, deoxyguanosine 5′-monophosphate, guanosine, and hypoxanthine-9-β-D-arabinofuranoside were found to be significantly decreased. Furthermore, it is well established that hypoxia-induced release of exosomes in tumor cells can significantly impact their secretion, composition, and function, potentially resulting in alterations in the proteins, lipids, and metabolites encapsulated within exosomes^24,25^. However, our research findings revealed no significant difference in serum exosome concentrations between the HC group and patients with HACE. This lack of distinction may be due to the diverse cell types present in the human body, as well as the various types of extracellular vesicles present in serum. Although tumor cells demonstrate increased exosome release under hypoxic conditions, it remains uncertain whether other cells may reduce exosome release under hypoxia, potentially explaining the consistent serum exosome concentrations observed.

Hypoxia-induced dysfunction of the blood‒brain barrier and subsequent cerebral edema are considered potential mechanisms underlying HACE^26^. During the early stages of cerebral ischemia and hypoxia, edema can develop through a process involving increased secretion of sodium, chloride, and permeable water, which cross the blood‒brain barrier from the blood into the brain^27^. Consequently, surrounding nerve cells rapidly swell as they absorb ions and water, resulting in cytotoxic edema^28^. Estrogen has been shown to protect against brain damage caused by ischemic stroke^29^. Specifically, estradiol acts on the endothelial cells of the blood‒brain barrier to reduce the formation of brain edema mediated by Na–K–Cl cotransporter proteins^30^. Our findings indicated that the levels of 17β-estradiol were upregulated during HACE. This finding suggested that, during the progression of HACE, the upregulation of estradiol in patients may play a critical role. Furthermore, we observed the differential expression of metabolites such as ( +)-camphoric acid, Ser-Ser, 20,26-dihydroxyecdysone, glycerophospho-N-arachidonoyl ethanolamine, choline, and acylglycine during HACE. Although these metabolites have rarely been reported in previous studies, they may play important roles in mediating signal transfer during HACE.

Based on the results of differentially abundant metabolite analysis, we conducted KEGG pathway enrichment analysis and identified several significantly affected metabolic pathways. These pathways included pathways related to energy metabolism regulation (such as purine metabolism, thermogenesis, and nucleotide metabolism), estrogen-related pathways (estrogen signaling pathway, GnRH signaling pathway, and GnRH pathway), cyclic nucleotide signaling pathways (cGMP-PKG signaling pathway and cAMP signaling pathway), and hormone synthesis and secretion (renin secretion, parathyroid hormone synthesis, secretion and action, and aldosterone synthesis and secretion). These abnormalities in metabolic pathways are primarily associated with ischemia-hypoxia, energy metabolism^31,32^, estrogen secretion^33^, and hormone secretion^34,35^ and have been previously identified in relevant studies on ischemia and hypoxia. Additionally, the analysis of regulatory networks of differentially abundant metabolites revealed the interconnectedness of these metabolites within the context of HACE. Therefore, our metabolomic data provide the basis for adopting a multitarget protection strategy for HACE.

We examined the associations between various factors and HACE scores using Pearson correlation analysis. The results revealed several significant associations, which provide valuable insights into the potential risk factors for HACE. We found an inverse relationship between stature and three metabolites: adenosine, guanosine, and hypoxanthine-9-β-D-arabinofuranoside. This finding suggested that individuals with a shorter stature may be at a greater risk for developing HACE. These metabolites have been previously implicated in oxygen deprivation^36^ and may play a role in the development of HACE. Additionally, we observed a negative correlation between BMI and deoxyguanosine 5′-monophosphate. This finding implies that individuals with a higher BMI may be less susceptible to HACE. The underlying mechanisms for this association need further investigation, but they may be related to the protective effects of higher BMI on oxygen deprivation. Furthermore, we found that the age of the subjects with HACE was positively correlated with LPE and pregnanetriol. This finding suggested that older individuals may be at a greater risk for developing HACE. LPE and pregnanetriol are substances involved in various physiological processes^37,38^, and their association with HACE warrants further research. It is important to note that correlation does not imply causation, and the directionality of the relationships observed in this study cannot be established. Additionally, the sample size of our study was relatively small, which may limit the generalizability of our findings. However, further studies with larger sample sizes and longitudinal designs are needed to confirm and expand upon our results.

## Conclusion

In conclusion, a metabolomic analysis of blood-derived exosomes from HACE patients highlighted significant alterations in metabolic pathways and identified potential biomarkers associated with this disease. The differential expression of metabolites linked to energy metabolism, hormone regulation, and hypoxia-induced dysfunction of the blood‒brain barrier provides a comprehensive understanding of the pathophysiology of HACE. The findings from this study could lead to the development of new strategies for early diagnosis and potential therapeutic targets for the management of HACE, contributing to improved clinical interventions in high-altitude environments. Further research with larger sample sizes and longitudinal designs is warranted to validate and expand on these findings for the advancement of HACE diagnosis and treatment.

### Supplementary Information

Supplementary Information.

Supplementary Figure 1.

Supplementary Figure 2.

Supplementary Table 1.

## Data Availability

All data generated or analysed during this study are included in this published article and its supplementary information files.

## Abbreviations

AMS: Acute mountain sickness
AUC: Area under curve
BMI: Body mass index
HACE: High-altitude cerebral edema
HC: Healthy control
KEGG: Kyoto Encyclopedia of Genes and Genomes
OPLS-DA: Orthogonal partial least squares discriminant analysis
PCA: Principal component analysis
ROC: Receiver operating characteristic
UPLC‒MS/MS: Ultraperformance liquid chromatography–tandem mass spectrometry
