# Supplementary material for: Identification of blood exosomal metabolomic profiling for high-altitude cerebral edema
Source: Sci Rep. 2024 May 21;14:11585. doi: 10.1038/s41598-024-62360-0 (PMC11109199; doi:10.1038/s41598-024-62360-0)
Supplement: Supplementary file 2 — Supplementary Figure 1. [file 41598_2024_62360_MOESM2_ESM.pdf]

Supplementary Figure 1

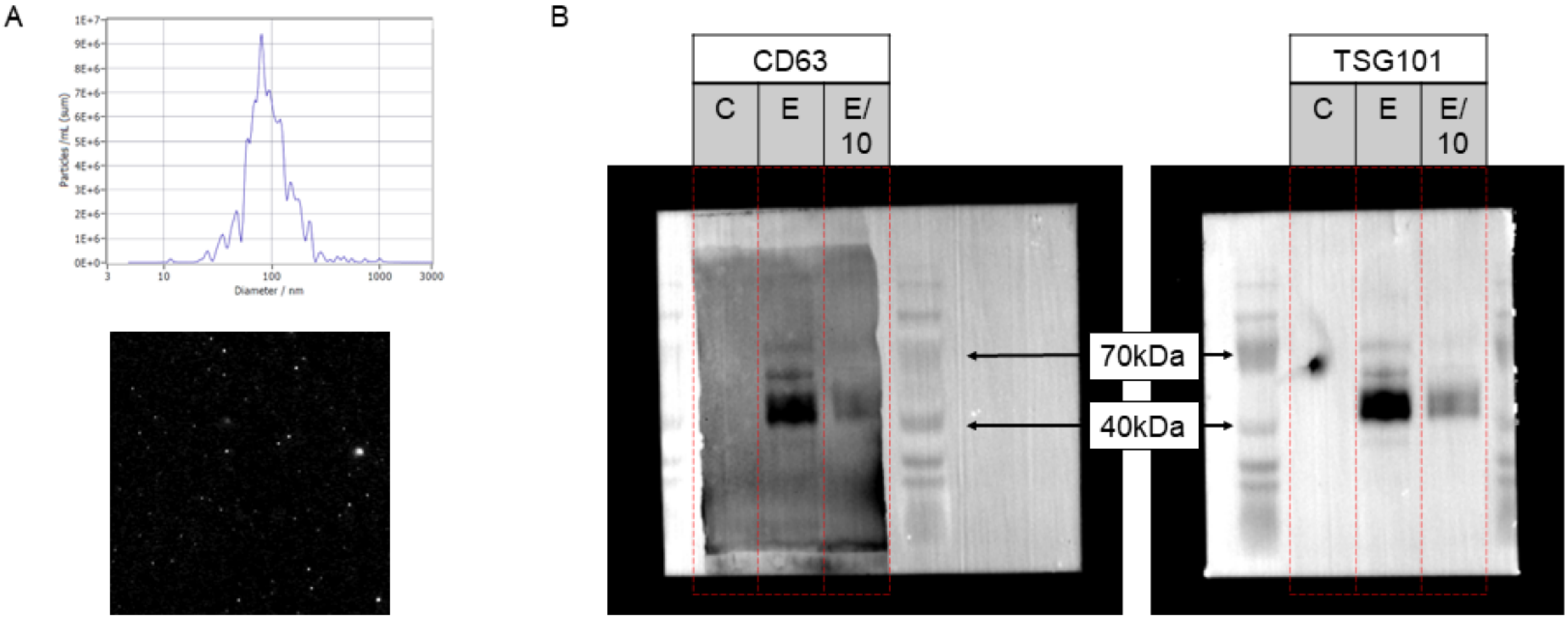

The results of exosome identification. (A) Nanoparticle tracking analysis (NTA) revealed that the peak particle size of the extracted exosomes was 86.7 nm, with over 98% falling within the range of 30-150 nm; (B) Western blot analysis confirmed the presence of specific exosome markers - CD63 and TSG101, C: PBS; E: Exosomes of serum; E/10: Dilute E ten times.
