# Supplementary material for: Identification of blood exosomal metabolomic profiling for high-altitude cerebral edema
Source: Sci Rep. 2024 May 21;14:11585. doi: 10.1038/s41598-024-62360-0 (PMC11109199; doi:10.1038/s41598-024-62360-0)
Supplement: Supplementary file 3 — Supplementary Figure 2. [file 41598_2024_62360_MOESM3_ESM.pdf]

Supplementary Figure 2

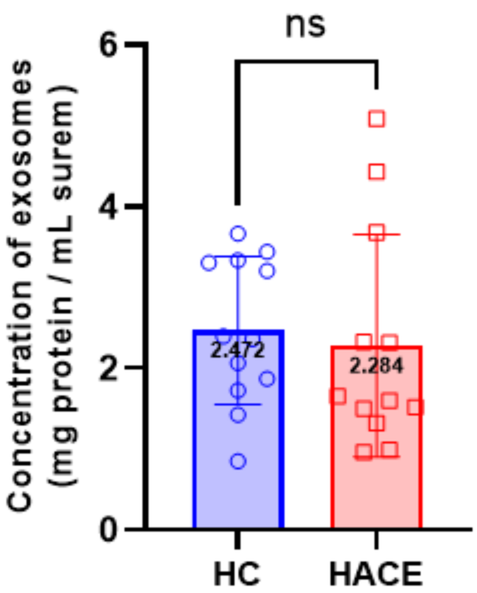

The concentration of exosomes in serum samples. There was no significant difference in serum exosome concentration between individuals with HC and HACE (2.472 ± 0.913 mg/mL, 2.284 ± 1.376 mg/mL; P = 0.6968).
