# Supplementary material for: Identification of blood exosomal metabolomic profiling for high-altitude cerebral edema
Source: Sci Rep. 2024 May 21;14:11585. doi: 10.1038/s41598-024-62360-0 (PMC11109199; doi:10.1038/s41598-024-62360-0)
Supplement: Supplementary file 4 — Supplementary Table 1. [file 41598_2024_62360_MOESM4_ESM.docx]

Supplementary Table 1 Blood parameters of the subjects.

| Blood  parameters | HC  (n=21) | HACE  (n=21) | P value | up / down |
| --- | --- | --- | --- | --- |
| WBC (10^9^/L) | 6.719 ± 1.540 | 9.752 ± 3.704 | 0.001278 | ↑ |
| RBC (10^12^/L) | 6.105 ± 0.776 | 5.167 ± 0.800 | 0.000405 | ↓ |
| HGB (g/L) | 178.619 ± 24.250 | 159.143 ± 27.657 | 0.019849 | - |
| PLT (10^9^/L) | 247.000 ± 66.309 | 191.048 ± 74.880 | 0.014225 | - |
| MPV (fL) | 10.381 ± 1.596 | 11.095 ± 0.889 | 0.080771 | - |
| PCT (%) | 0.259 ± 0.059 | 0.206 ± 0.074 | 0.013663 | - |
| MCV (fL) | 78.476 ± 30.657 | 95.000 ± 11.036 | 0.025293 | - |
| MCH (pg) | 29.238 ± 1.786 | 30.857 ±3.454 | 0.063571 | - |
| MCHC (g/L) | 323.857 ± 7.939 | 319.810 ± 16.449 | 0.315946 | - |
| NEUT% (%) | 56.819 ± 8.832 | 76.632 ± 7.265 | <0.000001 | ↑ |
| LYM% (%) | 33.486 ± 8.201 | 14.865 ± 5.394 | <0.000001 | ↓ |
| MONO% (%) | 7.386 ± 1.449 | 7.824 ± 2.920 | 0.541488 | - |
| EO% (%) | 2.143 ± 2.036 | 0.595 ± 1.964 | 0.016323 | - |
| BASO% (%) | 0.167 ± 0.128 | 0.095 ± 0.256 | 0.259258 | - |
| NEUT (10^9^/L) | 3.895 ± 1.331 | 7.548 ± 3.082 | 0.000012 | ↑ |
| LYMPH (10^9^/L) | 2.186 ± 0.524 | 1.395 ± 0.741 | 0.000273 | ↓ |
| MONO (10^9^/L) | 0.495 ± 0.175 | 0.762 ± 0.373 | 0.005093 | ↑ |
| EO (10^9^/L) | 0.138 ± 0.132 | 0.023 ± 0.056 | 0.000696 | ↓ |
| BAS0 (10^9^/L) | 0.011 ± 0.010 | 0.010 ± 0.037 | 0.909251 | - |
| HCT (%) | 55.048 ± 6.924 | 49.667 ± 7.812 | 0.023128 | - |
| CRP (mg/L) | 0.000 ± 0.000 (n=20)  <5.000 (n=1) | <5.000 (n=3)  31.813 ± 20.597 (n=15)  >200.000 (n=3) | <0.000001 | ↑ |

WBC: White blood cell count; RBC: Red blood count; HGB: Hemoglobin concentration; PLT: Platelet count; MPV: Mean platelet volume; PCT: Plateletcrit; MCV: Mean corpuscular volume; MCH: Mean corpuscular hemoglobin; MCHC: Mean corpuscular hemoglobin concentration; NEUT%: Neutrophil percentage; LYM%: Lymphocyte percentage; MONO%: Monocyte percentage; EO%: Eosinophil percentage; BASO%: Basophil percentage; NEUT: Neutrophil count; LYMPH: Lymphocyte count; MONO: Monocyte count; EO: Eosinophil count; BASO: Basophil count; HCT: Hematocrit; CRP: C-reactive protein.
